# The Pharmacokinetics/Pharmacodynamic Relationship of Durlobactam in Combination With Sulbactam in In Vitro and In Vivo Infection Model Systems Versus *Acinetobacter baumannii-calcoaceticus* Complex

**DOI:** 10.1093/cid/ciad096

**Published:** 2023-05-01

**Authors:** John P O’Donnell, Sujata M Bhavnani

**Affiliations:** Department of Drug Metabolism and Pharmacokinetics, Entasis Therapeutics, Waltham, Massachusetts, USA; Institute for Clinical Pharmacodynamics, Schenectady, New York, USA

**Keywords:** pharmacokinetics/pharmacodynamics, *Acinetobacter*, sulbactam, durlobactam, diazabicyclooctane

## Abstract

Sulbactam-durlobactam is a β-lactam/β-lactamase inhibitor combination currently in development for the treatment of infections caused by *Acinetobacter*, including multidrug-resistant (MDR) isolates. Although sulbactam is a β-lactamase inhibitor of a subset of Ambler class A enzymes, it also demonstrates intrinsic antibacterial activity against a limited number of bacterial species, including *Acinetobacter*, and has been used effectively in the treatment of susceptible *Acinetobacter*-associated infections. Increasing prevalence of β-lactamase–mediated resistance, however, has eroded the effectiveness of sulbactam in the treatment of this pathogen. Durlobactam is a rationally designed β-lactamase inhibitor within the diazabicyclooctane (DBO) class. The compound demonstrates a broad spectrum of inhibition of serine β-lactamase activity with particularly potent activity against class D enzymes, an attribute which differentiates it from other DBO inhibitors. When combined with sulbactam, durlobactam effectively restores the susceptibility of resistant isolates through β-lactamase inhibition. The present review describes the pharmacokinetic/pharmacodynamic (PK/PD) relationship associated with the activity of sulbactam and durlobactam established in nonclinical infection models with MDR *Acinetobacter baumannii* isolates. This information aids in the determination of PK/PD targets for efficacy, which can be used to forecast efficacious dose regimens of the combination in humans.

*Acinetobacter baumannii* is increasingly associated with serious nosocomial infections often accompanied by high rates of morbidity and mortality [1–4]. The majority of *A. baumannii* isolates are multidrug-resistant (MDR), resulting in limited treatment options [5]. Carbapenem-resistant *A. baumannii* has been identified as a global threat and has been designated as an urgent unmet medical need, requiring new treatment options [6, 7]. Sulbactam-durlobactam (SUL-DUR) is being developed for the treatment of *Acinetobacter baumannii-calcoaceticus* complex (ABC) infections, including those caused by MDR *A. baumannii*.

## CLINICAL USE OF SULBACTAM TO TREAT *ACINETOBACTER* INFECTIONS

Sulbactam is commonly used to treat *A. baumannii* infections due to its ability to inhibit penicillin-binding proteins (PBP1 and PBP3) in *Acinetobacter* spp., leading to cell death of the bacteria [8]. Clinically, *Acinetobacter* infections have been successfully treated with a high-dose 2:1 combination of ampicillin:sulbactam. Although some in vitro studies have suggested that ampicillin and sulbactam may act synergistically [9], clinical studies utilizing sulbactam administration alone suggest that the intrinsic activity of sulbactam is responsible for the observed efficacy of this combination versus *A. baumannii* infections encountered clinically [10]. As described below, these observations have also been supported in experimental infection models conducted in in vitro and in vivo studies [11–19].

## SULBACTAM EFFICACY OBSERVED IN ANIMAL INFECTION MODELS

In a neutropenic murine lung infection model, the efficacy of sulbactam was evaluated following administration of a 100-mg/kg dose every 3 hours (q3h) versus a susceptible *A. baumannii* isolate, SAN-94040, with a sulbactam minimum inhibitory concentration (MIC) of 0.5 µg/mL and a less-susceptible isolate, RCH-69, with a sulbactam MIC of 8 µg/mL [13]. The mice were rendered neutropenic and treatment was initiated 3 hours after infection of the lung. Treatment was conducted for 12 hours (4 doses of sulbactam administered intraperitoneally) and lungs were harvested 3 hours after the final dose to assess bacterial burden. Drug concentrations were determined systemically as well as in the lung. Serum concentrations of sulbactam were above the MIC for more than 3 hours vs SAN-94040 and 1.7 hours vs RCH-69. In the lung, concentrations of sulbactam exceeded the MIC for 4.8 and 1.3 hours for SAN-94040 and RCH-69, respectively. These treatments translated to end-of-treatment lung tissue burdens of 4.31 ± 0.19 and 6.4 ± 1.3 log_10_ colony-forming units (CFU)/g for SAN-94040 and RCH-69, respectively. Vehicle controls grew greater than 7 log_10_ CFU/g for both isolates. A bactericidal effect with a greater than 2-log_10_ CFU reduction from baseline was observed for SAN-94040 but not RCH-69. These findings were largely consistent with the pulmonary concentrations of sulbactam, which remained above the MIC of SAN-94040 for the entire dosing interval, but only above the MIC of RCH-69 for 43% of the dosing interval [13].

More recent studies have provided a rigorous assessment of the pharmacokinetic/pharmacodynamic (PK/PD) index most correlated with sulbactam activity and unbound exposure magnitude requirements to achieve PK/PD endpoints of net bacterial stasis and 1-, 2-, and 3-log_10_ CFU reductions from baseline in neutropenic murine thigh and lung infection models versus *A. baumannii* American Type Culture Collection (ATCC) 19606 [14]. This isolate was both sulbactam- and carbapenem-sensitive, with an MIC of 0.5 µg/mL for both sulbactam and imipenem. Dose fractionation was performed in both thigh and lung models over a dose range of 15 to 240 mg/kg administered at 2-, 3-, 4-, 6-, 12-, and 24-hour intervals. Comparison of Hill-type model fits describing the relationship between 24-hour change in log_10_ CFU/g burden and PK/PD indices of the time during 24 hours that unbound concentrations remain above the MIC (*f*T > MIC), the ratio of unbound area under the concentration-time curve over 0 to 24 hours to the MIC (*f*AUC_0–24_/MIC), and the ratio of unbound maximum concentration to the MIC (*f*C_max_/MIC) demonstrated correlation coefficients (*R*^2^) of 0.95, 0.60, and 0.37, respectively. Based on this analysis, the PK/PD index most closely associated with sulbactam activity was *f*T > MIC. The magnitude of sulbactam *f*T > MIC associated with achieving net bacterial stasis (no net change in bacterial counts over 24 hours of treatment) and 1-, 2-, and 3-log_10_ CFU reductions from baseline is summarized in Table 1. These magnitudes of *f*T > MIC were largely consistent with imipenem, which was used as a comparator in the study. Slightly higher potency was observed for sulbactam in the lung model compared with the thigh, with approximately 20% *f*T > MIC for a static effect and more than 60% and more than 40% *f*T > MIC for bactericidal effects in the thigh and lung, respectively.

**Table 1.** Magnitude of Sulbactam *f*T > MIC Associated With Net Bacterial Stasis, and 1-, 2-, and 3-Log_10_ CFU Reductions From Baseline Against *Acinetobacter baumannii* in Thigh and Lung Models

Bacterial Reduction Endpoint	Sulbactam *f*T > MIC (%) by Mouse Infection Model	
	Thigh	Lung
Net bacterial stasis	21.0	20.4
1-Log_10_ CFU reduction from baseline	32.9	24.5
2-Log_10_ CFU reduction from baseline	43.6	29.3
3-Log_10_ CFU reduction from baseline	57.3	37.3

Data from reference [14]. Abbreviations: CFU, colony-forming units; *f*T > MIC, time that unbound drug concentration remains above the minimum inhibitory concentration.

## SULBACTAM PK/PD TARGETS ESTABLISHED FROM IN VITRO INFECTION MODELS

**Figure 1.**
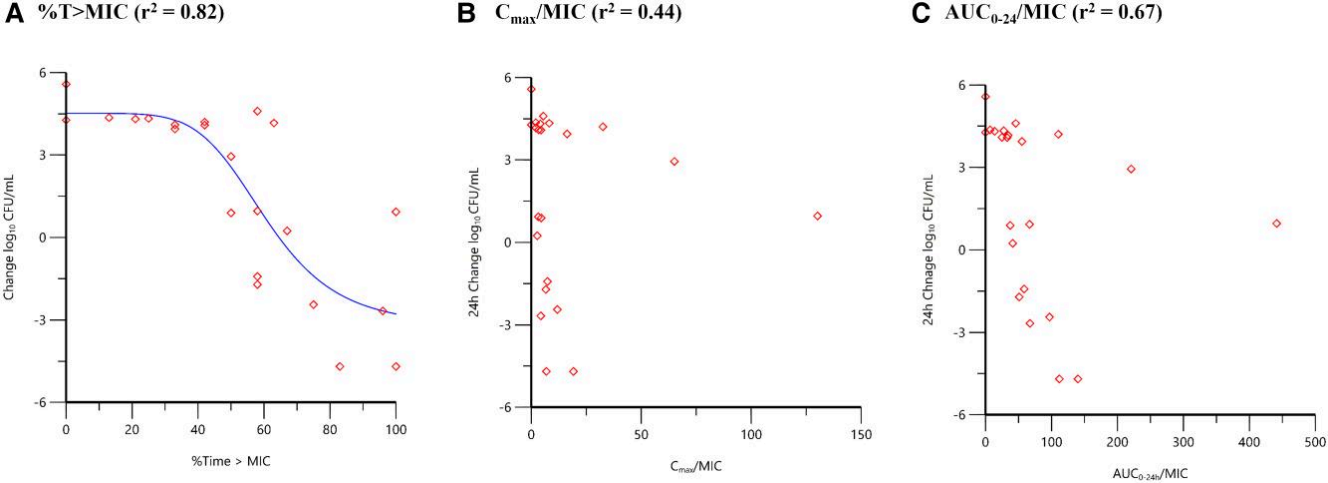
The 24-hour change in *Acinetobacter baumannii* Astra research collection (ARC)2058 (ADC-99 [N379S]; oxacillinase [OXA]-259) CFU/mL relative to sulbactam time (T) > MIC (*A*), C_max_/MIC (*B*), and AUC_0–24_/MIC (*C*) based on data from a hollow-fiber in vitro infection model (O'Donnell et al., Unpublished data): (*A*) %T > MIC (*r*^2^ = 0.82); (*B*) C_max_/MIC (*r*^2^ = 0.44); (*C*) AUC_0–24_/MIC (*r*^2^ = 0.67). Red diamond symbols represent PK parameter estimates derived from each dose fractionated regimen vs. the change in bacterial burden over 24 hours. The line represents the non-linear regression analysis of the data using the Hill equation. Abbreviations: AUC_0–24_, area under the concentration-time curve over 0 to 24 hours; CFU, colony-forming units; C_max_, maximum concentration; MIC, minimum inhibitory concentration.

The magnitude of *f*T > MIC associated with sulbactam efficacy was further investigated in in vitro dynamic model systems simulating the sulbactam component of 2:1 ampicillin:sulbactam human PK exposures in an evaluation of MDR *A. baumannii* isolates ranging in sulbactam MIC values of 2 to 32 µg/mL [15]. Free-drug concentrations of sulbactam from 3 g ampicillin/sulbactam (2 g/1 g) every 6 hours (q6h) (0.5-hour infusion) and 9 g ampicillin/sulbactam (6 g/3 g) every 8 hours (q8h) (3-hour infusion) were evaluated in a one-compartment in vitro PK/PD model over 24 hours to determine the net change in log_10_ CFU/mL from baseline as well as the area under the bactericidal curve (AUBC). Both the 3-g q6h and the 9-g q8h regimens provided exposure consistent with 100% *f*T > MIC compared with the MDR isolate, ACB 35 (MIC = 2 µg/mL), and resulted in sustained bactericidal activity over the 24 hours of the experiment. The lower dose of 3 g demonstrated minimal efficacy versus ACB 32 (MIC = 32 µg/mL), with an observed *f*T > MIC of only 5.9%. Nearly a 1-log_10_ CFU reduction from baseline was achieved versus ACB 31 and ACB 33 (MIC = 16 µg/mL), with an observed *f*T > MIC of approximately 29%. Results for the higher 9-g dose regimen were variable but still not effective compared with ACB 32 (*f*T > MIC of 50.7%), although higher AUBCs were achieved across all the isolates relative to the lower 3-g dose regimen. It was suggested that higher *f*T > MIC exposure may be needed in vitro to maintain efficacy or a higher concentration of sulbactam itself to circumvent higher β-lactamase production associated with the MDR isolates [15]. Additional studies were undertaken using a hollow-fiber in vitro infection model to reconfirm the PK/PD index associated with sulbactam efficacy and the magnitude of such an index required for various levels of bacterial reduction (O'Donnell et al., Unpublished data). High magnitudes of sulbactam *f*T > MIC associated with net bacterial stasis and 1- and 2-log_10_ CFU/mL reductions from baseline were observed in dose-fractionation studies, whereas lower sulbactam exposure magnitudes of sulbactam *f*T > MIC associated with these endpoints were observed in in vivo models. Consistent with results from previous studies, however, *f*T > MIC was identified as the PK/PD index most closely associated with the activity of sulbactam (Figure 1). The reason for the higher magnitudes of *f*T > MIC in the hollow-fiber in vitro system is still unknown. However, the lower molecular weight cutoff (5 kD) of the hollow-fiber cartridge may trap and accumulate β-lactamases, which we have confirmed through nitrocefin assay of cartridge contents (J. O'Donnell 2023, unpublished data). This potentially serves as a sink for sulbactam, reducing the amount of unbound drug available to interact with PBPs as well as reducing the targeted concentration of the β-lactam in the system—a phenomenon observed by other investigators [16, 17]. To avoid this, further studies were carried out to determine the magnitude of sulbactam *f*T > MIC associated with efficacy in in vitro chemostat and in vivo neutropenic murine infection models, in which the concentration of β-lactamase does not confound interpretation of study results (O'Donnell et al., Unpublished data).

In vivo studies completed to support the PK/PD understanding of sulbactam and durlobactam were carried out using neutropenic murine thigh and lung infection models and contemporary *A. baumannii* clinical isolates with relevant resistance determinants spanning a broad range of MIC values (O'Donnell et al., Unpublished data). Initial dose-ranging studies based on mouse PK and sulbactam susceptibility (with and without durlobactam) suggested that a 4:1 dose ratio of sulbactam:durlobactam from 2.5:0.625 mg/kg to 80:20 mg/kg administered every 3 to 6 hours would be sufficient to explore PK/PD relationships for efficacy [18]. Dose titration in a 4:1 ratio dosing of sulbactam:durlobactam was completed versus MDR *A. baumannii* strain ARC3486 (OXA-66, OXA-72, Temoneria [TEM]-1, acinetobacter-derived cephalosporinase [ADC]-30), which had a sulbactam MIC of 32 µg/mL or greater. In the presence of a 4-µg/mL concentration of durlobactam, the sulbactam MIC versus ARC3486 was reduced to 0.5 µg/mL. Initial tissue burden at the time of treatment was 6.36 log_10_ CFU/g in the thigh model and 7.40 log_10_ CFU/g in the lung model. In vehicle (no treatment) controls, colonies grew approximately 2 log_10_ CFU from baseline over 24 hours. At the top dose of 80:20 sulbactam:durlobactam q3h, a greater than 2 log_10_ CFU/g reduction was achieved over 24 hours in both models, with a clear dose–response observed across the dose range. An additional study was performed in the neutropenic murine thigh infection model utilizing a 15-mg/kg dose of sulbactam q3h across all dose arms and titrating the durlobactam dose from 1.25 to 50 mg/kg in combination. The 15-mg/kg dose was identified in PK studies to be consistent with unbound sulbactam concentrations exceeding an MIC (of sulbactam alone) of 0.5 µg/mL for 50% of the dosing interval, an exposure associated with achieving a 1-log_10_ CFU or greater reduction from baseline across the infection models completed in vitro and in vivo (Table 2) (O'Donnell et al., Unpublished data). Sulbactam administered alone at a dose of 15 mg/kg q3h was ineffective and nearly equivalent to the vehicle (no treatment) control. This observation was expected, as the MIC of sulbactam alone versus ARC3486 was 32 µg/mL or greater. The addition of durlobactam resulted in a clear dose-dependent reduction from baseline bacterial burden (log_10_ CFU/g), with near maximal activity observed at a dose of 15:5 mg/kg sulbactam:durlobactam administered q3h. Similar studies using a higher fixed dose of sulbactam to cover a higher SUL-DUR MIC of 4 µg/mL were also completed [19]. The MDR *A. baumannii* strain used in these investigations, ARC5955 (TEM-1, ADC-82, OXA-23, and OXA-66), had a sulbactam MIC of 64 µg/mL, but activity was restored to an MIC of 4 µg/mL with the addition of durlobactam. A dose of 75 mg/kg q3h was evaluated across all dose arms in a neutropenic murine thigh infection model, with increasing doses of durlobactam added from 12.5 to 200 mg/kg. The sulbactam dose of 75 mg/kg was selected to achieve *f*T ≥ 4 µg/mL for greater than 50% of the dosing interval. Neither sulbactam nor durlobactam administered by themselves at 75 and 50 mg/kg, respectively, q3h were effective, with 2.5- and 2.2-log_10_ CFU/g growth observed over 24 hours. Net bacterial stasis was achieved when 12.5 mg/kg of durlobactam was added to sulbactam (75 mg/kg) and a 1-log_10_ CFU reduction from baseline was observed when the durlobactam dose was increased to a dose of 50 mg/kg. Another half-log_10_ CFU/g reduction from baseline was achieved when a 200-mg/kg dose of durlobactam was added to sulbactam (75 mg/kg).

**Table 2.** Magnitude of Sulbactam *f*T > MIC Associated With 1- and 2-Log_10_ CFU Reductions From Baseline and EC_80_ Based on Data From In Vitro and In Vivo Studies

Model	No. of Isolates		Sulbactam *f*T > MIC (%) by Endpoint		
			CFU Reduction From Baseline		EC_80_
		*r* ^2^	1-Log_10_	2-Log_10_	
In vitro hollow fiber^a^	1	0.82	71.5	82.0	93.6
In vivo thigh (4:1 dose)^b^	6	0.83	29.9	38.2	44.1
In vivo lung (4:1 dose)^c^	5	0.89	41.2	53.5	>100
Mean ± SD	…	…	47.5 ± 21.5	57.9 ± 22.2	68.9 ± 35.0

Data from reference (O'Donnell et al., Unpublished data). EC_80_, the percentage of time required for unbound concentrations to remain above the MIC in order to achieve 80% of the maximum activity.Abbreviations: CFU, colony-forming units; *f*T > MIC, time that unbound drug concentration remains above the minimum inhibitory concentration; SD, standard deviation

Sulbactam-sensitive isolate *A. baumannii* ARC2058.

Single pooled dataset Hill-type fitting across 6 *A. baumannii* isolates; sulbactam:durlobactam, 4:1.

Single pooled dataset Hill-type fitting across 5 *A. baumannii* isolates; sulbactam:durlobactam, 4:1.

## DURLOBACTAM PK/PD TARGETS ESTABLISHED FROM A ONE-COMPARTMENT IN VITRO INFECTION MODEL

Having established a PK/PD target of approximately 50% *f*T > MIC for sulbactam, further in vitro studies were pursued using a one-compartment chemostat model to determine the PK/PD index and magnitude of such an index associated with durlobactam activity (O'Donnell et al., Unpublished data). Several recent publications have investigated the PK/PD of contemporary β-lactamase inhibitors, reporting that the PK/PD index most closely associated with activity was either unbound concentrations exceeding a critical threshold (C_T_) or unbound AUC_0–24_/MIC. It has been suggested that the type of inhibition observed biochemically with the inhibitor may provide insight into the optimum PK/PD index associated with its activity [20, 21]. The PK/PD of the β-lactamase inhibitor tazobactam, which demonstrates a relatively slow on rate and slow off rate appears to be driven by *f*T > C_T_ [22]. By contrast, nearly irreversible inhibitors such as vaborbactam [23] and relebactam [24] may benefit from a time-exposure and concentration dependency associated typically with mechanism-based inactivators demonstrating greater affinity and rapid on rates [21]. For these inhibitors, the AUC may be a more relevant parameter associated with the inhibitor–enzyme interaction and target occupancy [25]. Durlobactam inhibition of class D β-lactamases, which are highly prevalent in *A. baumannii* isolates, has been shown to be particularly potent with *k*_inact_/*K*_i_ values nearly 1000-fold higher than avibactam [18, 26] and a partition ratio of nearly 1 against the vast majority of β-lactamases [27]. With these types of covalent interactions, inhibition would be expected to increase over time with exposure as opposed to reaching equilibrium. Based on these observations, one might expect *f*AUC/MIC to be the PK/PD driver for durlobactam.

Studies in the one-compartment system were carried out using a single MDR *A. baumannii* isolate, ARC5081, which demonstrated a sulbactam MIC of 16 µg/mL and an MIC of 4 µg/mL for sulbactam in the presence of 4 µg/mL of durlobactam (O'Donnell et al., Unpublished data). Initial dose-ranging studies with sulbactam administered q6h at clinically equivalent exposures of 2 g administered every 6 hours combined with durlobactam administered q6h with a durlobactam *f*AUC_0–24_ range of 18.5 to 591 µg · hour/mL were performed prior to dose fractionation with durlobactam. Dose fractionation was then completed using durlobactam *f*AUC_0–24_ of 13.9, 55.8, 111, and 222 µg · hour/mL administered every 6, 12, or 24 hours. For all studies, sulbactam and durlobactam were administered into the system via a 3-hour infusion. At sulbactam 2 g q6h, the time above the SUL-DUR MIC of 4 µg/mL exceeded 50% of the dosing interval. The relationships between each of durlobactam AUC_0–24_, C_max_, and the percentage of time that durlobactam concentrations were above the C_T_ values ranging from 0.5 to 2 µg/mL and the 24-hour change in log CFU/g were evaluated using Hill-type models and nonlinear least-squares regression. Fitting of the data demonstrated that free time above a critical threshold (*f*T > C_T_) of 0.75 µg/mL was most highly correlated to the observed activity of durlobactam when administered in combination with sulbactam. While time-dependent activity is associated with the PK/PD of durlobactam, the half-life of the compound precludes a meaningful analysis of the dose-fractionation data to establish PK/PD targets clinically. When only the q6h and q12h data were considered, however, AUC_0–24_ was considered a more informative PK/PD index, with data scattered equally across the Hill-type function and a clear maximum effect observed (O'Donnell et al., Unpublished data). Because a q6h regimen of sulbactam is required clinically to achieve its PK/PD target of 50% *f*T > MIC, a q6h durlobactam regimen was considered. Based on the Hill-type model fit of *f*AUC_0–24_ versus 24-hour change log_10_ CFU/mL and administration of durlobactam q6h, 1- and 2-log_10_ CFU reductions from baseline were associated with *f*AUC_0–24_ of 30.5 µg · h/mL and 134 µg · h/mL, respectively, versus ARC5081. Using the modal SUL-DUR MIC of 4 µg/mL for ARC5081, these exposures correspond to *f*AUC_0–24_:MIC ratios of approximately 10 and 30 for 1- and 2-log_10_ CFU reductions from baseline, respectively. In summary, while the PK/PD of durlobactam was shown to demonstrate time-dependent activity in vitro, *f*AUC_0–24_/MIC was shown to correlate to activity using the q6h and q12h dosing, likely due to the short half-life of the compound.

## SULBACTAM AND DURLOBACTAM PK/PD TARGET MAGNITUDES ESTABLISHED IN VIVO

Pharmacokinetic/pharmacodynamic analyses for both sulbactam and durlobactam were performed utilizing data for multiple *A. baumannii* isolates evaluated using neutropenic murine thigh and lung infection models. Isolates were selected with the goal to evaluate a broad range of MIC values below and above the projected breakpoint of 4 µg/mL. Although MDR bacteria can exhibit a number of resistance mechanisms, in the case of carbapenem-resistant *Acinetobacter*, the prevailing resistance mechanism is β-lactamase production. It follows that bacteria with higher MIC values likely have higher expression levels of β-lactamase and, thus, would require higher β-lactamase inhibitor exposure to effectively restore wild-type MIC distribution of the β-lactam. Thus, a direct translational relationship should exist between inhibitor exposure and the MIC. This has been shown in the evaluation of the β-lactamase inhibitor tazobactam used in combination with ceftolozane, for which co-modeling of 7 isolates was performed by normalizing the %*f*T > C_T_ by the MIC, where the C_T_ was the product of the ceftolozane -tazobactam MIC for each individual isolate multiplied by 0.5 [28]. Thus, pooled and co-modeled data spanning a broad range of MIC were considered together to arrive at a single exposure target directly related to in vitro susceptibility of the isolate.

A sulbactam-sensitive *A. baumannii* isolate, ARC2058, was used in neutropenic murine thigh and lung infection models to compare the magnitude of sulbactam *f*T > MIC associated with efficacy in the thigh and lung for sulbactam treatment alone. In the lung model, the mean *f*T > MIC magnitudes required for a 1-log_10_ and 2-log_10_ CFU reduction from baseline and the exposure required to reach 80% of maximum activity (EC_80_) were 37.8%, 50.1%, and 68.5%, respectively. In the thigh model, mean %*f*T > MIC magnitudes required for a 1-log_10_ and 2-log_10_ CFU reduction from baseline and the EC_80_ were 20.5%, 31.5%, and 47.0%, respectively. These magnitudes were close to exposures associated with efficacy of sulbactam in human clinical studies [10, 29–31] when considering human PK parameters for sulbactam [32, 33].

**Figure 2.**
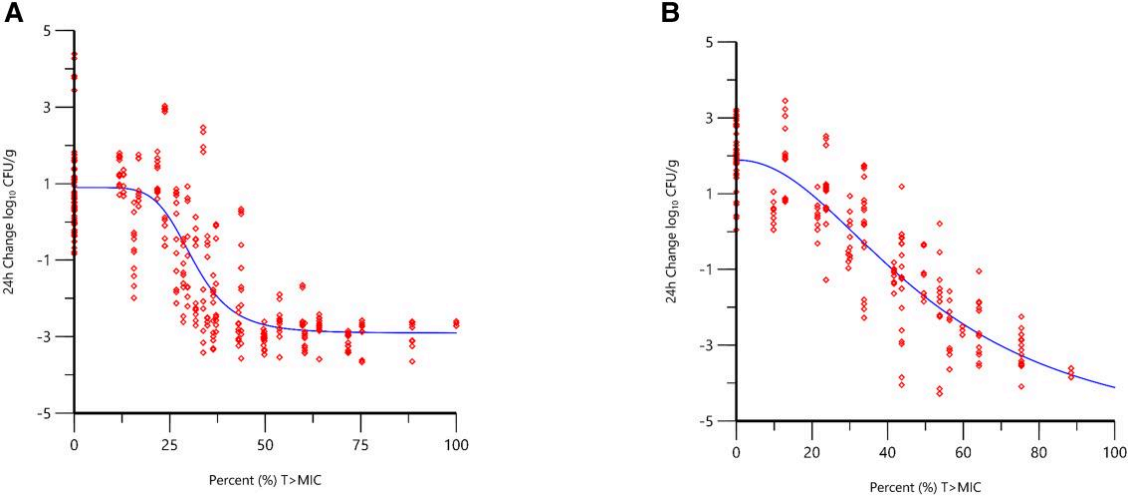
Change in bacterial burden (log_10_ CFU/g) over 24 hours vs %*f*T > MIC of sulbactam when administered alone and combined with durlobactam based on data from neutropenic murine thigh (*r*^2^ = 0.83, n = 6 isolates) (*A*) and lung (*r*^2^ = 0.89, n = 5 isolates) (*B*) infection models (O'Donnell et al., Unpublished data). Red diamond symbols represent PK parameter estimates derived from each dose fractionated regimen vs. the change inbacterial burden over 24 hours. The line represents the non-linear regression analysis of the data using the Hill equation. Abbreviations: CFU, colony-forming units; *f*T > MIC, time that unbound drug concentration remains above the minimum inhibitory concentration; MIC, minimum inhibitory concentration.

Up to 5 recent MDR *A. baumannii* clinical isolates, for which the β-lactamase genotypes had previously been determined by whole-genome sequencing (ARC3484, ARC3486, ARC5079, ARC5081, and ARC5091), were evaluated in addition to ARC2058 in dose–response studies conducted using neutropenic murine thigh and lung infection models. These studies were carried out to determine the %*f*T > MIC magnitudes required by sulbactam when administered in the presence of durlobactam (O'Donnell et al., Unpublished data). Co-modeling of the %*f*T > MIC sulbactam exposure response data across multiple MDR isolates and ARC2058 was performed utilizing the data obtained from both neutropenic thigh and lung models incorporating 4:1 sulbactam:durlobactam administration (Figure 2). The 24-hour change in CFU/g from the initiation of therapy versus *f*T > MIC for the pooled dataset of all isolates used in each model was fit to a Hill-type function, and the magnitude of sulbactam *f*T > MIC targets associated with efficacy are summarized in Table 2. Magnitudes of *f*T > MIC were higher in the lung model compared with the thigh model, with *f*T > MIC of 41.2% versus 29.9% for 1-log_10_ CFU reduction from baseline and 53.5% versus 38.2% for a 2-log_10_ CFU reduction from baseline in the lung and thigh models, respectively.

**Figure 3.**
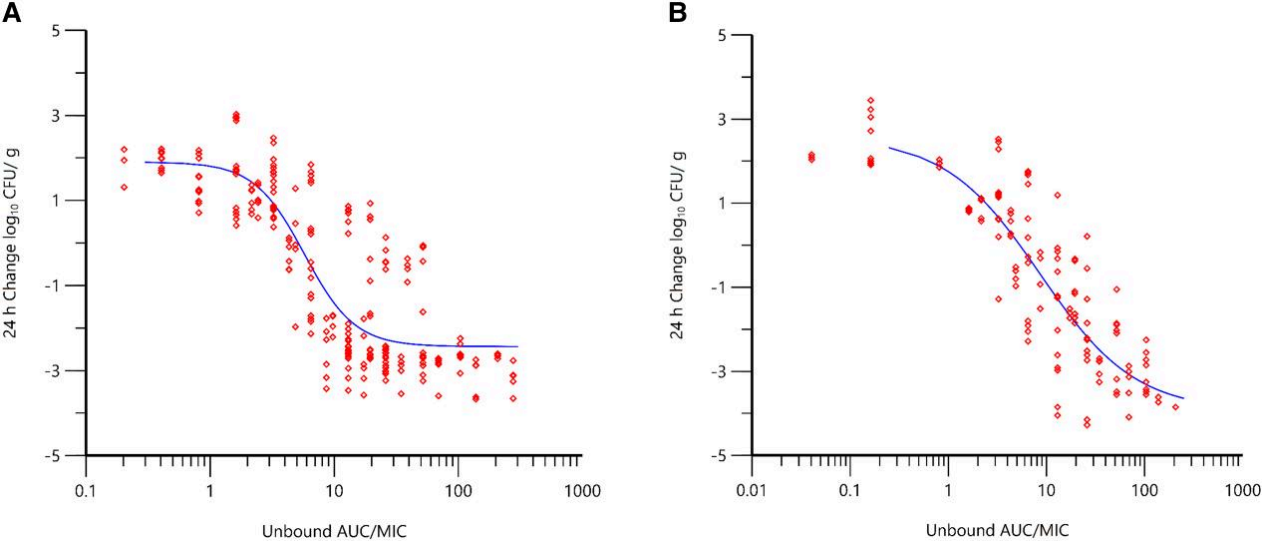
Change in bacterial burden (log_10_ CFU/g) over 24 hours vs *f*AUC_0–24_/MIC of durlobactam when administered in combination with sulbactam based on data from neutropenic murine thigh (*r*^2^ = 0.86, n = 5 isolates) (*A*) and lung (*r*^2^ = 0.91 n = 4 isolates) (*B*) infection models (O'Donnell et al., Unpublished data). Red diamond symbols represent PK parameter estimates derived from each dose fractionated regimen vs. the change inbacterial burden over 24 hours. The line represents the non-linear regression analysis of the data using the Hill equation. Abbreviations: CFU, colony-forming units; *f*AUC_0–24_/MIC, ratio of unbound area under the concentration-time curve over 0 to 24 hours to the minimum inhibitory concentration.

Co-modeling of the durlobactam PK/PD data across multiple MDR isolates and normalizing the AUC_0–24_ by SUL-DUR MIC in the thigh and lung models resulted in Hill-type model fits shown in Figure 3. The SUL-DUR MIC values of these MDR isolates ranged from 1 to 4 µg/mL. Correlations (*r*^2^) of 0.86 and 0.91 were observed for thigh and lung models, respectively. For studies incorporating a fixed dose of sulbactam (to keep the *f*T > MIC of sulbactam consistent throughout the exposure range of durlobactam), a correlation of 0.82 was observed. The magnitudes of durlobactam *f*AUC/MIC associated with efficacy are summarized in Table 3. Unbound AUC_0–24_/MIC magnitudes were generally consistent in both lung and thigh models as well as in the chemostat model, with *f*AUC_0–24_/MIC magnitudes of 10 and 30 associated with 1-log_10_ and 2-log_10_ CFU reductions from baseline, respectively.

**Table 3.** Magnitude of Durlobactam *f*AUC_0–24_/MIC Associated With 1- and 2-Log_10_ CFU Reductions From Baseline and EC_80_ Based on Data From In Vitro and In Vivo Studies

Model	No. of Isolates	*r* ^2^	Durlobactam *f*AUC_0–24_/MIC by Endpoint		
			CFU Reduction From Baseline		EC_80_
			1-Log_10_ CFU Reduction	2-Log_10_ CFU Reduction	
In vitro chemostat^a^	1	0.87	7.6	33.4	NC
In vivo thigh (4:1 dose)^b^	5	0.86	8.0	16.0	15.1
In vivo lung (4:1 dose)^c^	4	0.91	10.6	22.4	78.6
In vivo thigh (fixed SUL dose)^d^	6	0.82	7.5	31.8	38.2
Mean ± SD	…	…	8.4 ± 1.5	25.9 ± 8.2	44.0 ± 32.1

Data from reference (O'Donnell et al., Unpublished data). PK-PD analysis of 24-hour net CFU reduction vs *f*AUC_0–24_/MIC. EC_80_, the percentage of time required for unbound concentrations to remain above the MIC in order to achieve 80% of the maximum activity. Abbreviations: CFU, colony-forming units; *f*AUC_0–24_/MIC, ratio of unbound area under the concentration-time curve over 0 to 24 hours to the minimum inhibitory concentration; NC, not calculated; PK/PD, pharmacokinetic/pharmacodynamic; SD, standard deviation; SUL, sulbactam.

SUL exposure at >50% *f*T > MIC.

SUL mean exposure of 32.9% to 43.5% *f*T > MIC; sulbactam:durlobactam, 4:1.

SUL mean exposure of 43.9% to 81.5% *f*T > MIC; sulbactam:durlobactam, 4:1.

SUL exposure range of 18.2% to 52.7% *f*T > MIC; sulbactam dose held constant.

## SULBACTAM-DURLOBACTAM PK/PD TARGETS FOR EFFICACY

Taken collectively, the in vitro and in vivo data support a 1-log_10_ CFU reduction over 24 hours when sulbactam 50% *f*T > MIC and durlobactam *f*AUC_0–24_/MIC of 10 in the combination were achieved. This level of bactericidal activity has been suggested to correlate to clinical outcome and efficacy in patients with hospital-acquired pneumonia and bacteremia [34–36].

## CONCLUSIONS

These data confirm %T > MIC to be the PK/PD index that best describes sulbactam efficacy, with unbound exposures above the MIC for 50% of the dosing interval being associated with a 1-log_10_ CFU reduction from baseline based upon data from in vitro dose-fractionation studies. For durlobactam, %T > C_T_ of 0.75 µg/mL was identified as the PK/PD index associated with efficacy against a single MDR isolate using a one-compartment in vitro infection model. Targeting AUC_0–24_/MIC with divided (q6h) dosing, however, was also highly correlated to activity across the MIC range of isolates, with a ratio of approximately 10 being associated with achieving a 1-log_10_ CFU reduction from baseline when a sulbactam 50% *f*T > MIC target associated with this endpoint was also achieved.

Studies completed in vivo also demonstrated that the SUL-DUR combination is effective in treating *A. baumannii* in neutropenic murine thigh and lung infection models, achieving at least a 1-log_10_ CFU reduction from baseline over 24 hours of dosing against all MDR isolates evaluated. The magnitudes of the PK/PD indices associated with efficacy for each agent in each of these infection models were relatively consistent between models. An unbound AUC:MIC ratio of approximately 10 for durlobactam and 50% *f*T > MIC for sulbactam for a 1-log_10_ CFU reduction from baseline were supported by in vivo and in vitro studies and are likely to correlate with clinical efficacy. The results of analyses assessing the probability of attaining these PK/PD targets based on human exposures in the epithelial lining fluid support the proposed clinical dose of 1 g/1 g sulbactam/durlobactam administered via a 3-hour infusion q6h to treat patients with *A. baumannii* isolates with MIC values of 4 µg/mL or less [37]. Moreover, the most recent in vitro surveillance data demonstrate an SUL-DUR MIC_90_ of 2 µg/mL for 5032 ABC isolates, thereby supporting the goal for covering a potentiated MIC of 4 µg/mL [38]. Based on these results, SUL-DUR may represent a potentially compelling treatment option over current standard of care, which includes high-dose ampicillin-sulbactam administered in combination with tetracyclines, polymyxin B, extended infusions of meropenem, or cefiderocol [5].

## Notes

***Acknowledgments.*** The authors acknowledge the support from AstraZeneca Pharmaceuticals Waltham, MA, and NeoSome Life Sciences, Billerica, MA, for their support and completion of the in vivo murine infection models.

***Financial support.*** This work was sponsored by Entasis Therapeutics, Inc., a wholly owned subsidiary of Innoviva, Inc. The authors did not receive any fees for authorship.

***Supplement sponsorship.*** This article appears as part of the supplement “Sulbactam-durlobactam, a Targeted β-lactam/β-lactamase Inhibitor, for MDR *Acinetobacter*,” sponsored by Entasis Therapeutics Inc., a wholly owned subsidiary of Innoviva, Inc.

## References

[1] Lemos EV , de la Hoz FP, Einarson TR, et al Carbapenem resistance and mortality in patients with *Acinetobacter baumannii* infection: systematic review and meta-analysis. Clin Microbiol Infect 2014; 20:416–23. 24131374 10.1111/1469-0691.12363

[2] Cai B , Echols R, Magee G, et al Prevalence of carbapenem-resistant gram-negative infections in the United States predominated by *Acinetobacter baumannii* and *Pseudomonas aeruginosa*. Open Forum Infect Dis 2017; 4:ofx176. 10.1093/ofid/ofx176 PMC5629822 29026867

[3] Zilberberg MD , Nathanson BH, Sulham K, Fan W, Shorr AF. Multidrug resistance, inappropriate empiric therapy, and hospital mortality in *Acinetobacter baumannii* pneumonia and sepsis. Crit Care 2016; 20:221. 27417949 10.1186/s13054-016-1392-4 PMC4946176

[4] Clark NM , Zhanel GG, Lynch JP 3rd . Emergence of antimicrobial resistance among *Acinetobacter* species: a global threat. Curr Opin Crit Care 2016; 22:491–9. 27552304 10.1097/MCC.0000000000000337

[5] Tamma PD , Aitken SL, Bonomo RA, Mathers AJ, van Duin D, Clancy CJ. Infectious Diseases Society of America guidance on the treatment of AmpC β-lactamase-producing enterobacterales, carbapenem-resistant *Acinetobacter baumannii*, and *Stenotrophomonas maltophilia* infections. Clin Infect Dis 2022; 74:2089–114. 34864936 10.1093/cid/ciab1013

[6] Centers for Disease Control and Prevention, National Center for Emerging and Zoonotic Infections Diseases, Division of Healthcare Quality Promotion . COVID-19: U.S. impact on antimicrobial resistance, special report 2022. Atlanta, GA: US Department of Health and Human Services, Centers for Disease Control and Prevention, 2022.

[7] World Health Organization . Media Centre. News release. WHO publishes list of bacteria for which new antibiotics are urgently needed, 2017. Available at: http://www.who.int/mediacentre/news/releases/2017/bacteria-antibioticsneeded/. Accessed 1 February 2023.

[8] Penwell WF , Shapiro AB, Giacobbe RA, et al Molecular mechanisms of sulbactam antibacterial activity and resistance determinants in *Acinetobacter baumannii*. Antimicrob Agents Chemother 2015; 59:1680–9. 25561334 10.1128/AAC.04808-14 PMC4325763

[9] Labia R , Morand A, Lelievre V, Mattioni D, Kazmierczak A. Sulbactam: biochemical factors involved in its synergy with ampicillin. Rev Infect Dis 1986; 8:S496–502. 3025996 10.1093/clinids/8.supplement_5.s496

[10] Corbella X , Ariza J, Ardanuy C, et al Efficacy of sulbactam alone and in combination with ampicillin in nosocomial infections caused by multiresistant *Acinetobacter baumannii*. J Antimicrob Chemother 1998; 42:793–802. 10052904 10.1093/jac/42.6.793

[11] Obana Y , Nishino T. *In-vitro* and *in-vivo* activities of sulbactam and YTR830H against *Acinetobacter calcoaceticus*. J Antimicrob Chemother 1990; 26:677–82. 1964157 10.1093/jac/26.5.677

[12] Rodríguez-Hernández MJ , Cuberos L, Pichardo C, et al Sulbactam efficacy in experimental models caused by susceptible and intermediate *Acinetobacter baumannii* strains. J Antimicrob Chemother 2001; 47:479–82. 11266426 10.1093/jac/47.4.479

[13] Wolff M , Joly-Guillou ML, Farinotti R, Carbon C. *In vivo* efficacies of combinations of β-lactams, β-lactamase inhibitors, and rifampin against *Acinetobacter baumannii* in a mouse pneumonia model. Antimicrob Agents Chemother 1999; 43:1406–11. 10348761 10.1128/aac.43.6.1406 PMC89287

[14] Yokoyama Y , Matsumoto K, Ikawa K, et al Pharmacokinetic/pharmacodynamic evaluation of sulbactam against *Acinetobacter baumannii* in *in vitro* and murine thigh and lung infection models. Int J Antimicrob Agents 2014; 43:547–52. 24796218 10.1016/j.ijantimicag.2014.02.012

[15] Housman ST , Hagihara M, Nicolau DP, Kuti JL. *In vitro* pharmacodynamics of human-simulated exposures of ampicillin/sulbactam, doripenem and tigecycline alone and in combination against multidrug-resistant *Acinetobacter baumannii*. J Antimicrob Chemother 2013; 68:2296–304. 23710070 10.1093/jac/dkt197

[16] Bulitta JB , Hope WW, Eakin AE, et al Generating robust and informative nonclinical *in vitr*o and *in vivo* bacterial infection model efficacy data to support translation to humans. Antimicrob Agents Chemother 2019; 63:e02307–18. 30833428 10.1128/AAC.02307-18 PMC6496039

[17] Drusano GL , Bonomo RA, Bahniuk N, et al Resistance emergence mechanism and mechanism of resistance suppression by tobramycin for cefepime for *Pseudomonas aeruginosa*. Antimicrob Agents Chemother 2012; 56:231–42. 22005996 10.1128/AAC.05252-11 PMC3256024

[18] Durand-Réville TF , Guler S, Comita-Prevoir J, et al ETX2514 is a broad-spectrum β-lactamase inhibitor for the treatment of drug-resistant gram-negative bacteria including *Acinetobacter baumannii*. Nat Microbiol 2017; 2:17104. 28665414 10.1038/nmicrobiol.2017.104

[19] Barnes MD , Kumar V, Bethel CR, et al Targeting multidrug-resistant *Acinetobacter* spp. sulbactam and the diazabicyclooctenone β-lactamase inhibitor ETX2514 as a novel therapeutic agent. mBio 2019; 10:e00159–19. 30862744 10.1128/mBio.00159-19 PMC6414696

[20] Bhagunde P , Zhang Z, Racine F, et al A translational pharmacokinetic/pharmacodynamic model to characterize bacterial kill in the presence of imipenem-relebactam. Int J Infect Dis 2019; 89:55–61. 31479762 10.1016/j.ijid.2019.08.026

[21] Ambrose PG , Lomovskaya O, Griffith DC, Dudley MN, VanScoy B. β-Lactamase inhibitors: what you really need to know. Curr Opin Pharmacol 2017; 36:86–93. 29096172 10.1016/j.coph.2017.09.001

[22] Nicasio AM , VanScoy BD, Mendes RE, et al Pharmacokinetics-pharmacodynamics of tazobactam in combination with piperacillin in an *in vitro* infection model. Antimicrob Agents Chemother 2016; 60:2075–80. 26787689 10.1128/AAC.02747-15 PMC4808219

[23] Griffith DC , Sabet M, Tarazi Z, Lomovskaya O, Dudley MN. Pharmacokinetics/pharmacodynamics of vaborbactam, a novel β-lactamase inhibitor, in combination with meropenem. Antimicrob Agents Chemother 2018; 63:e01659–18. 30397063 10.1128/AAC.01659-18 PMC6325214

[24] Blizzard TA , Chen H, Kim S, et al Discovery of MK-7655, a β-lactamase inhibitor for combination with Primaxin®. Bioorg Med Chem Lett 2014; 24:780–5. 24433862 10.1016/j.bmcl.2013.12.101

[25] Strelow JM . A perspective on the kinetics of covalent and irreversible inhibition. SLAS Discov 2017; 22:3–20. 27703080 10.1177/1087057116671509

[26] Ehmann DE , Jahić H, Ross PL, et al Kinetics of avibactam inhibition against class A, C, and D β-lactamases. J Biol Chem 2013; 288:27960–71. 23913691 10.1074/jbc.M113.485979 PMC3784710

[27] Shapiro AB , Gao N, Jahić H, Carter NM, Chen A, Miller AA. Reversibility of covalent, broad-spectrum serine β-lactamase inhibition by the diazabicyclooctenone ETX2514. ACS Infect Dis 2017; 3:833–44. 28835096 10.1021/acsinfecdis.7b00113

[28] Vanscoy B , Mendes RE, McCauley J, et al Pharmacological basis of β-lactamase inhibitor therapeutics: tazobactam in combination with ceftolozane. Antimicrob Agents Chemother 2013; 57:5924–30. 24041895 10.1128/AAC.00656-13 PMC3837916

[29] Cisneros JM , Reyes MJ, Pachón J, et al Bacteremia due to *Acinetobacter baumannii*: epidemiology, clinical findings, and prognostic features. Clin Infect Dis 1996; 22:1026–32. 8783704 10.1093/clinids/22.6.1026

[30] Levin AS , Levy CE, Manrique AE, Medeiros EA, Costa SF. Severe nosocomial infections with imipenem-resistant *Acinetobacter baumannii* treated with ampicillin/sulbactam. Int J Antimicrob Agents 2003; 21:58–62. 12507838 10.1016/s0924-8579(02)00276-5

[31] Betrosian AP , Frantzeskaki F, Xanthaki A, Georgiadis G. High-dose ampicillin-sulbactam as an alternative treatment of late-onset VAP from multidrug-resistant *Acinetobacter baumannii*. Scand J Infect Dis 2007; 39:38–43. 17366011 10.1080/00365540600951184

[32] Meyers BR , Wilkinson P, Mendelson MH, Walsh S, Bournazos C, Hirschman SZ. Pharmacokinetics of ampicillin-sulbactam in healthy elderly and young volunteers. Antimicrob Agents Chemother 1991; 35:2098–101. 1759832 10.1128/aac.35.10.2098 PMC245332

[33] Soto E , Shoji S, Muto C, Tomono Y, Marshall S. Population pharmacokinetics of ampicillin and sulbactam in patients with community-acquired pneumonia: evaluation of the impact of renal impairment. Br J Clin Pharmacol 2014; 77:509–21. 24102758 10.1111/bcp.12232 PMC4371533

[34] Bulik CC , Bhavnani SM, Hammel J, et al Relationship between regulatory approval and pharmacokinetic-pharmacodynamic target attainment: focus on community- and hospital-acquired pneumonia. [abstract A-295]. Presented at 53rd Interscience Conference on Antimicrobial Agents and Chemotherapy (Denver, CO). Washington, DC: American Society for Microbiology, 2013.

[35] Trang M , Dudley MN, Bhavnani SM. Use of Monte Carlo simulation and considerations for PK-PD targets to support antibacterial dose selection. Curr Opin Pharmacol 2017; 36:107–13. 29128853 10.1016/j.coph.2017.09.009

[36] Ambrose PG . Antibacterial drug development program successes and failures: a pharmacometric explanation. Curr Opin Pharmacol 2017; 36:1–7. 28688237 10.1016/j.coph.2017.06.002

[37] Bhavnani SM , Rubino CM, Hammel JP, et al Population pharmacokinetic (PPK), pharmacokinetic/pharmacodynamic (PTA), and clinical pharamcockinetic/pharmacodynamic (PK/PD) analyses for sulbactam-durlobactam (SUL-DUR) to support dose selection for the treatment of *Acinetobacter baumannii-calcoaceticus* complex (ABC) [abstract LB2306]. Presented at IDWeek 2022. Washington, DC: Infectious Diseases Society of America, 2022.

[38] Karlowsky JA , Hackel MA, McLeod SM, Miller AA. *In vitro* activity of sulbactam-durlobactam against global isolates of *Acinetobacter baumannii-calcoaceticus* complex collected from 2016 to 2021. Antimicrob Agents Chemother 2022; 66:e0078122. 10.1128/aac.00781-22 PMC9487466 36005804

